# Molecular Interaction and Evolution of Jasmonate Signaling With Transport and Detoxification of Heavy Metals and Metalloids in Plants

**DOI:** 10.3389/fpls.2021.665842

**Published:** 2021-04-14

**Authors:** Xuan Chen, Wei Jiang, Tao Tong, Guang Chen, Fanrong Zeng, Sunghoon Jang, Wei Gao, Zhen Li, Michelle Mak, Fenglin Deng, Zhong-Hua Chen

**Affiliations:** ^1^Collaborative Innovation Center for Grain Industry, College of Agriculture, Yangtze University, Jingzhou, China; ^2^Central Laboratory, Zhejiang Academy of Agricultural Science, Hangzhou, China; ^3^Department of Life Sciences, Pohang University of Science and Technology, Pohang, South Korea; ^4^State Key Laboratory of Crop Stress Adaptation and Improvement, Henan University, Kaifeng, China; ^5^School of Agriculture, Jinhua Polytechnic, Jinhua, China; ^6^School of Science, Western Sydney University, Penrith, NSW, Australia; ^7^Hawkesbury Institute for the Environment, Western Sydney University, Penrith, NSW, Australia

**Keywords:** jasmonic acid, plant evolution, comparative genomics, cadmium, arsenic, detoxification

## Abstract

An increase in environmental pollution resulting from toxic heavy metals and metalloids [e.g., cadmium (Cd), arsenic (As), and lead (Pb)] causes serious health risks to humans and animals. Mitigation strategies need to be developed to reduce the accumulation of the toxic elements in plant-derived foods. Natural and genetically-engineered plants with hyper-tolerant and hyper-accumulating capacity of toxic minerals are valuable for phytoremediation. However, the molecular mechanisms of detoxification and accumulation in plants have only been demonstrated in very few plant species such as *Arabidopsis* and rice. Here, we review the physiological and molecular aspects of jasmonic acid and the jasmonate derivatives (JAs) in response to toxic heavy metals and metalloids. Jasmonates have been identified in, limiting the accumulation and enhancing the tolerance to the toxic elements, by coordinating the ion transport system, the activity of antioxidant enzymes, and the chelating capacity in plants. We also propose the potential involvement of Ca^2+^ signaling in the stress-induced production of jasmonates. Comparative transcriptomics analyses using the public datasets reveal the key gene families involved in the JA-responsive routes. Furthermore, we show that JAs may function as a fundamental phytohormone that protects plants from heavy metals and metalloids as demonstrated by the evolutionary conservation and diversity of these gene families in a large number of species of the major green plant lineages. Using ATP-Binding Cassette G (ABCG) transporter subfamily of six representative green plant species, we propose that JA transporters in Subgroup 4 of ABCGs may also have roles in heavy metal detoxification. Our paper may provide guidance toward the selection and development of suitable plant and crop species that are tolerant to toxic heavy metals and metalloids.

## Introduction

Naturally occurring toxic heavy metals and metalloids are usually dispersed around the world at low levels; however, large quantities of them have been released into global ecosystems through various anthropogenic activities such as mining, smelting, and other industrial and agricultural activities (Tomar et al., 2015; Zhao et al., 2015; Deng et al., 2021). Heavy metals and metalloids are usually elements with a density higher than 5 g cm^−3^ (Tchounwou et al., 2012). Heavy elements displaying potential arrest on organisms growth, development, and productivity are classified as toxic heavy metals and metalloids (Järup, 2003; Clemens and Ma, 2016; Nguyen et al., 2021; Paithankar et al., 2021). The major threats to human health and the environment from heavy metals and metalloids are attributing to exposure to arsenic (As), cadmium (Cd), lead (Pb), and mercury (Hg; Järup, 2003). For example, a slow poisoning by Cd or As exposure may lead to overall increases in mortality and a range of diseases (Clemens and Ma, 2016). Ingestion is one of the major routes for human exposure to hazardous minerals, while the food chain represents the primary source (Rojas-Rueda et al., 2021); therefore, we need to reduce the accumulation of the toxic minerals in the edible organs of plants.

In the plant kingdom, the phytotoxicity from Pb and Hg ranks upper most in the list of hazardous metals, while high concentration of the essential mineral copper (Cu) displaying higher toxicity than those of As and Cd. The median toxic concentrations of Pb, Hg, Cu, Cd, and As that reduces plant growth in solution culture are estimated as 0.30, 0.47, 2.0, 5.0, 9.0 μM, respectively (Kopittke et al., 2010). In addition to being environmental pollutants, other elements such as chromium (Cr), nickel (Ni), and other trace metals also cause considerable harm to humans and plants (Kan et al., 2021). Therefore, high accumulation of toxic, nonessential minerals and excessive doses of essential minerals should be significantly reduced from plant-based food and animal feed. Strategies such as identifying new crop species and generating new crop cultivars with lower accumulating activity (Deng et al., 2018, 2019; Huang et al., 2020; Huang and Zhao, 2020; Zhao and Wang, 2020), using natural or genetically engineered hyperaccumulating plants for phytoremediation have been proposed for the future food safety (Deng et al., 2021).

As a primary defense-signaling hormone, jasmonates coordinate growth, and defense responses to developmental and various environmental cues (Ahmad et al., 2016; Howe et al., 2018; Yu et al., 2019). Since the initial identification of methylester of JA (MeJA) as an odor of *Jasminum grandiflorum* flowers, major progress on the biosynthesis, metabolism, and modes of action in response to stresses and the developmental process of jasmonates have been elucidated. Additionally, Jasmonates signal plant defenses against biotic stressors such as insects and necrotrophic fungi (Wasternack and Song, 2017). Temporal and spatial regulation of jasmonate signaling is crucial in the elegant control of plant growth (Jin and Zhu, 2017), while JA biosynthesis for plant defense upon insect attack is rapidly activated (Yan et al., 2018a). Herbivory defenses are usually regulated *via* Ca^2+^ signaling [e.g., glutamate receptor-like proteins (GLRs), calmodulins (CaMs)] for wound signals transmission from leaf-to-leaf and activate JA-mediated plant defense (Nguyen et al., 2018), which subsequently inactivate the JA biosynthesis repressor complex consisting of AtJAV1-AtJAZ8-AtWRKY51 through interaction with AtJAV1 (Yan et al., 2018a). In this review, we mainly focus on the overview of gene families involved in the JA biosynthesis and signal transduction and their potential link to the tolerance of toxic metals and metalloids in plants.

Membrane-localized mineral transporters in the cellular and long-distance allocation of minerals play a significant role in the detoxification and accumulation of toxic heavy metals and metalloids in plants (Tomar et al., 2015; Clemens and Ma, 2016; Lindsay and Maathuis, 2017; Yamaji and Ma, 2017; Deng et al., 2019, 2021; Hu et al., 2020; Sharma et al., 2020; Zhao and Wang, 2020; Liu et al., 2021; Tang and Zhao, 2021). For example, *Arabidopsis* (*Arabidopsis thaliana*) Iron Regulated Transporter 1 (AtIRT1), a members of the ZIP (zinc-regulated transporter) family, is the primary transporter for Cd uptake (Lux et al., 2011), while the translocation of Cd from roots (R) to shoots (SH) is mediated by two root pericycle-localized Heavy Metal ATPases (HMAs), AtHMA2, and AtHMA4 (Hussain et al., 2004; Mills et al., 2005). The homolog AtHMA3 is localized on the tonoplast and responsible for Cd sequestration from the cytosol into the vacuole (Chao et al., 2012). On the other hand, proteins in the Natural Resistance Associated Macrophage Proteins family, AtNramp3 and AtNramp4, release Cd from vacuolar lumen to cytoplasm (Pottier et al., 2015).

Although the vital functions of phytohormones, in their prospective regulatory networks, in sensing the stress generated by toxic heavy metals and metalloids have been demonstrated, evidence linking JA to the physiological responses to toxic elements are still lacking (Chmielowska-Bak et al., 2014; Deng et al., 2020). The long-distance translocation and cellular mobility of toxic minerals can be regulated by signaling molecules through membrane transport systems. For example, ABA can inhibit Cd and Arsenate [As(V)] uptake through ABI5-MYB49-IRT1/HIPPs and WRKY6-PHT1;1 network, respectively (Hu et al., 2020). Additionally, ABA promotes the chelation and compartmentation of heavy metals through ABA-responsive transcriptional factors (Hu et al., 2020). Moreover, increasing pieces of evidence demonstrated the involvement of jasmonates consisting of JA and its derivatives such as jasmonoyl-l-isoleucine (JA-Ile) and methyl-JA in the detoxification and transport of toxic mineral stress (Maksymiec et al., 2005; Chen et al., 2014; Zhao et al., 2016; Wang et al., 2018; Bali et al., 2019; Lei et al., 2020b). Some regulatory mechanisms of jasmonates in response to toxic heavy metals and metalloids have been revealed in model plants (Lei et al., 2020b) and major cereals such as rice (*Oryza sativa*) (Yu et al., 2012; Azeem, 2018; Mousavi et al., 2020), but the evolutionary origin linking JA signaling and plant tolerance to toxic elements is less studied in other green plants including green algae, bryophytes, lycophytes, ferns, and gymnosperms (Chen et al., 2017; Adem et al., 2020; Deng et al., 2021). These regulatory mechanisms in algae and early-divergent plants such as ferns may contribute to the removal of heavy metals from water and soil (Ma et al., 2001; Cheng et al., 2019; Manara et al., 2020).

Physiological roles of the mineral transporters (Adem et al., 2020; Hu et al., 2020; Deng et al., 2021) and jasmonates signaling (Howe and Yoshida, 2019; Monte et al., 2019) may vary among plant species, but heavy metals and metalloids elevate endogenous JA levels in many plants. The growing number of plant genomes (Kersey, 2019) and transcriptomes (One Thousand Plant Transcriptomes Initiative, 2019) has enabled the comparative evolutionary analysis of key gene families relevant to the resistance of element contaminates in green plants, which will be helpful in searching for potential mitigation practices. Here, we summarize molecular interaction between jasmonate signaling and heavy metals detoxification in plants. We also trace the origin and evolution of the core components linking JA signaling and tolerance to toxic metals and metalloids in plants. For topics on JA signaling and heavy metal and metalloid tolerance in plants, some recent reviews are suggested (Clemens and Ma, 2016; Howe et al., 2018; Wasternack and Feussner, 2018; Deng et al., 2020, 2021; Zhao and Wang, 2020).

## Overview of Jasmonates Mediated Regulatory Network

### The Biosynthesis and Metabolism of Jasmonates

Jasmonates are synthesized from α-linolenic acid (α-LeA) through the octadecanoid pathway (Wasternack and Feussner, 2018). α-LeA is released from galactolipids of chloroplast membranes by chloroplast-targeted galactolipases encoded by *Defective in Anther Dehiscence 1* (*DAD1*) and its homologs including *Dongle* (*DGL*) and *DAD1-like lipases* (*DALL*) (Ishiguro et al., 2001; Hyun et al., 2008; Ruduś et al., 2014). The position C-13 of α-LeA is converted to 13S-hydroperoxyoctadecatrienoic acid (13-HPOT) by the plastid-localized 13-Lipoxygenases (13-LOXs). Subsequently, the generation of oxophytodienoic acid [OPDA; *cis*-(+)-12-oxophytodienoic acid] from 13-HPOT is catalyzed by a two-step reaction required allene oxide synthases (AOSs; Laudert et al., 1996; Sivasankar et al., 2000) and allene oxide cyclases (AOCs; Ziegler et al., 1997, 2000). The transport of OPDA from plastids to peroxisomes is essential for the next steps including OPDA reduction and ß-oxidation of the carboxylic acid side chain in higher plants (Wasternack and Song, 2017; Howe and Yoshida, 2019). The efflux of OPDA from plastids is mediated by outer chloroplast envelope-localized JASSY (Guan et al., 2019), while the import of OPDA into the peroxisomes is partially reliant on peroxisomal ABC-transporter 1 (PXA1) in *Arabidopsis* (AtABCD1; Theodoulou et al., 2005). JASSY is a chloroplast membrane-localized 12-oxophytodienoic acid (OPDA) transporter, while AtABCD1 is a member of the D-subgroup of the ATP-Binding Cassette (ABC) transporter family. The conversion of OPC-8 [3-oxo-2-(2-pentenyl)-cyclopentane-1-octanoic acid] from OPDA is produced by peroxisomal OPDA reductases (OPRs; Schaller et al., 2000; Stintzi and Browse, 2000). OPC-8:CoA ligase 1 (OPCL1) is required for the formation of OPC-CoA ester (Koo et al., 2006; Kienow et al., 2008) and then three rounds of ß-oxidation are catalyzed by acyl-CoA oxidases (ACXs; Schilmiller et al., 2007), the multifunctional proteins (MFPs), and l-3-ketoacyl-CoA thiolases (KATs; Cruz Castillo et al., 2004; Li et al., 2005; Wasternack and Feussner, 2018). The generated (+7)-iso-JA [also known as (*3R,7S*)-JA or JA] is then transported to the cytoplasm for further modifications.

Among the JA derivatives in higher plants, the conjugated (+)-7-*iso*-JA-Ile (JA-Ile) is the most biologically active form (Han, 2017). The conjugation between (+)-7-*iso*-JA and isoleucine is catalyzed by JA conjugate synthase (JA-amino acid synthetase, JAR1), a member of the GH3 family (AtGH3.11; Staswick and Tiryaki, 2004). The deconjugation is cleaved by IAA-Ala-Resistant 3 (IAR3) and IAA-Leu Resistant-like 6 (ILL6) of the ILR1-like amidohydrolase (IAH; Widemann et al., 2013; Koo, 2018). Furthermore, the oxidative inactivation from JA-Ile to 12-hydroxy-JA-Ile (12OH-JA-Ile) is mediated by the cytochrome P450 subfamily of CYP94 proteins, including CYP94B1, CYP94B2, CYP94B3, and CYP94C1 (Koo et al., 2011; Heitz et al., 2012; Bruckhoff et al., 2016). Recently, Jasmonate-Induced Oxygenases (JOXs) have been identified as the enzymes responsible for the hydroxylation and inactivation of the JA (Caarls et al., 2017). In addition to JA-Ile, methyl jasmonate (MeJA) is another well-known form of jasmonates in stress response and development in most land plants. The methyl esterification form is produced by the activity of jasmonic acid carboxyl methyltransferase (JMT; Seo et al., 2001). Apart from the conjugation, methylation, esterification, hydroxylation, and carboxylation, JA is also able to be modified by sulfation and O-Glycosylation, which may be required for transport and storage (Wasternack and Song, 2017; Wasternack and Feussner, 2018).

### The Perception and Core Components of Jasmonates Signaling

The fine-tuning of JA-signaling is regulated by synergistic and antagonistic activities of various signaling components. The active JA-Ile is perceived *via* a complex of co-receptors, consisting of Coronatine Insensitive 1 (COI1) and jasmonate-ZIM domain proteins (JAZs; Xie et al., 1998; Xu et al., 2002; Yan et al., 2009; Sheard et al., 2010; Yan et al., 2018b). In *Arabidopsis*, the F-box protein COI1 is able to physically interact with CULLIN 1 (AtCUL1), RING-box1 (AtRbx1), Skp1-like proteins (AtASK1), or (AtASK2) to assemble Skp1/Cullin/F-box protein ubiquitin E3 ligase complex SCF^COI1^ (Xu et al., 2002). JAZs function as negative regulators of the transcription factors such as MYC-related transcriptional activators (MYC, a subgruop of transcriptional factor belonging to the basic helix-loop-helix (bHLH) proteins; Kazan and Manners, 2013) and jasmonate-associated VQ motif gene 1 (JAV1; Hu et al., 2013a). In the absence of active JAs, MYCs are repressed by JAZs and the interacting partners [TOPLESS (TPL), TPL-related proteins (TRPs)], and Novel Interactor of JAZ (NINJA; Pauwels et al., 2010). In the presence of JA-Ile, JAZs are ubiquitinated and degraded, thus released MYCs can activate the expression of JA responsive genes and trigger downstream responses (Pauwels et al., 2010). Notably, the SCF^COI1^-JAZ-MYC complex relays JAs-specific regulatory signals to generate transcriptional regulation through Mediator 25 (MED25) (Çevik et al., 2012; Chen et al., 2012). Upon degradation of JAZ repressors, MED25 interacts with MYC2 and recruits Histone Acetyltransferase 1 (HAC1) as well as Pol II to the promoters of MYC2 target genes, and thereby activate their expression (An et al., 2017). The activity of MYC2 is then inhibited by Jasmonate-Associated MYC2-like (AtJAMs) in *Arabidopsis* (Sasaki-Sekimoto et al., 2013; Liu et al., 2019; Wasternack, 2019). Additionally, proteins such as *Arabidopsis* Histone Deacetylase 6 (AtHDA6; Wu et al., 2008) and AtHDA19 (Zhou et al., 2005) have been shown to play a role in regulating gene expression involved in JA signaling.

*Arabidopsis* Jasmonate Transporter 1 (AtJAT1) is a member of the ABC transporters (AtABCG16), which controls efflux of JA-Ile into the nuclear and cellular regions (Li et al., 2017b). In addition, four homologs of AtJAT1 including AtJAT2~5 (AtABCG1/6/20/2) have been identified as the candidates of jasmonate transporters (Wang et al., 2019a). Among them, AtJAT2 is localized in the peroxisomes and may facilitate the export of JA into the cytosol, while the plasma membrane-localized AtJAT3/4/5 may be involved in the subcellular distribution of jasmonates (Wang et al., 2019a). Furthermore, the long-distance transport of JAs from wounded to undamaged leaves seems to be mediated by *Arabidopsis* Glucosinolate Transporter 1 (AtGTR1), belonging to a member of Nitrate Transporter 1/Peptide Transporter Family (NPF) and encoding by *AtNPF2.10* (Saito et al., 2015; Ishimaru et al., 2017). Downstream signaling and physiological responses to jasmonates are transduced by the JAZ–transcription factor modules in plants (Pauwels and Goossens, 2011; Qi et al., 2015; Hu et al., 2017; Jin and Zhu, 2017; Howe et al., 2018; Yan et al., 2018b; Howe and Yoshida, 2019). For example, subgroup IIIb basic helix–loop–helix proteins (bHLHs) including Inducer of CBF Expression 1 (ICE1) and ICE2 form complexes with JAZs to promote cold acclimation responses in both *Arabidopsis* (Hu et al., 2013b) and banana (Zhao et al., 2013). In rice, OsJAZ9 interacts with transcription factor, OsbHLH062, to alter ion homeostasis (Wu et al., 2015), while Rice Salt Sensitive3 (OsRSS3) mediates the interaction between OsJAZ8/9/11 and OsbHLH089/094, leading to reprogramming root growth in high salinity environments through JA-responsive pathways (Toda et al., 2013).

## Jasmonates Contribute to Plant Tolerance to Toxic Heavy Metals and Metalloids

Growing evidence demonstrates the positive roles of JA in the detoxification of and tolerance to toxic heavy metals and metalloids (Yan et al., 2013; Per et al., 2016; Zhao et al., 2016; Li et al., 2017a; Wang et al., 2018; Bali et al., 2019; Lei et al., 2020b; Mousavi et al., 2020). Numerous physiological studies demonstrate that endogenous jasmonates levels in plants rapidly elevate when exposed to heavy metals and metalloids (Rakwal et al., 1996; Maksymiec et al., 2005; Rodríguez-Serrano et al., 2006; Ronzan et al., 2019; Lei et al., 2020b). The dynamics of jasmonates accumulation in the leaves of *Arabidopsis* exposed to high Cu or Cd display a biphasic character. An initial, rapid increment, of JA levels occurs and reaches a maximum at 7 h after the Cu or Cd treatments, followed by a rapid decrease during the next 7 h. The highest levels of JA induced by Cu and Cd were 4-fold and approximately 7-fold of the control, respectively. Then, a phase of repeated but slow incremental increases of JA content was observed in the leaves (Maksymiec et al., 2005). JA concentration in the roots of *Arabidopsis* is also elevated following the treatment of Cd for 6 h, the content is higher than control at 3 days but shows no significant difference after 7 days (Lei et al., 2020b). Increased levels of JAs are observed in Cu- or Cd-treated runner bean (*Phaseolus coccineus*; Maksymiec et al., 2005), Cd-treated pea (*Pisum sativum*; Rodríguez-Serrano et al., 2006), Ni-exposed woody shrub *Daphne jasmine* (Wiszniewska et al., 2018), and Cu-affected rice leaves (Rakwal et al., 1996). Moreover, the increased production of JA in hyperaccumulator *Noccaea (Thlaspi) praecox* by Cd is dependent on mechanically puncturing or fungal infection (Llugany et al., 2013), indicating the positive roles of Cd-induced JA in metal hyperaccumulators under abiotic and biotic stresses. However, it was found that Zn-induced while salicylic acid (SA) pathway (not JA pathway) is activated when the metal hyperaccumulator plant *Noccaea caerulescens* inoculated with *Pseudomonas syringae* (Fones et al., 2013). For metalloids, JA-Ile content in rice roots is rapidly increased by the application of arsenite [As(III)] for 8 h (Ronzan et al., 2019).

Consistently, exogenous JAs are widely employed to alleviate the plant growth inhibition caused by heavy metals and metalloids. For example, 0.25, 0.5, and 1 μM MeJA alleviates As(III) toxicity in rice (Mousavi et al., 2020; Verma et al., 2020), the elongation of rice roots pretreated with 0.5–5 μM JA mediated significantly less inhibition of root elongation by As(V) than non-treated plants (Wang et al., 2018). Exogenous application of 25 μM JA improved tolerance of rapeseed (*Brassica napus*) to Cd toxicity (Ali et al., 2018), while 1 μM MeJA partially regulated As(III) stress in oilseed (*B. napus*; Farooq et al., 2016), Ni stress in maize (*Zea mays*; Azeem, 2018) and soybean (*Glycine max*; Sirhindi et al., 2016), Cd toxicity in mustard (Per et al., 2016), faba bean (*Vicia faba*; Ahmad et al., 2017), and Solanaceae (*Solanum nigrum*; Yan et al., 2015b). The combined action of Cd and Cu in *Avicennia marina* can be partially diminished by the addition of JA or MeJA (Yan et al., 2015a). Taken together, these observations indicate that the elevated JA induced by toxic metals is a common stress responsive mechanism in different plant species.

**Figure 1:**
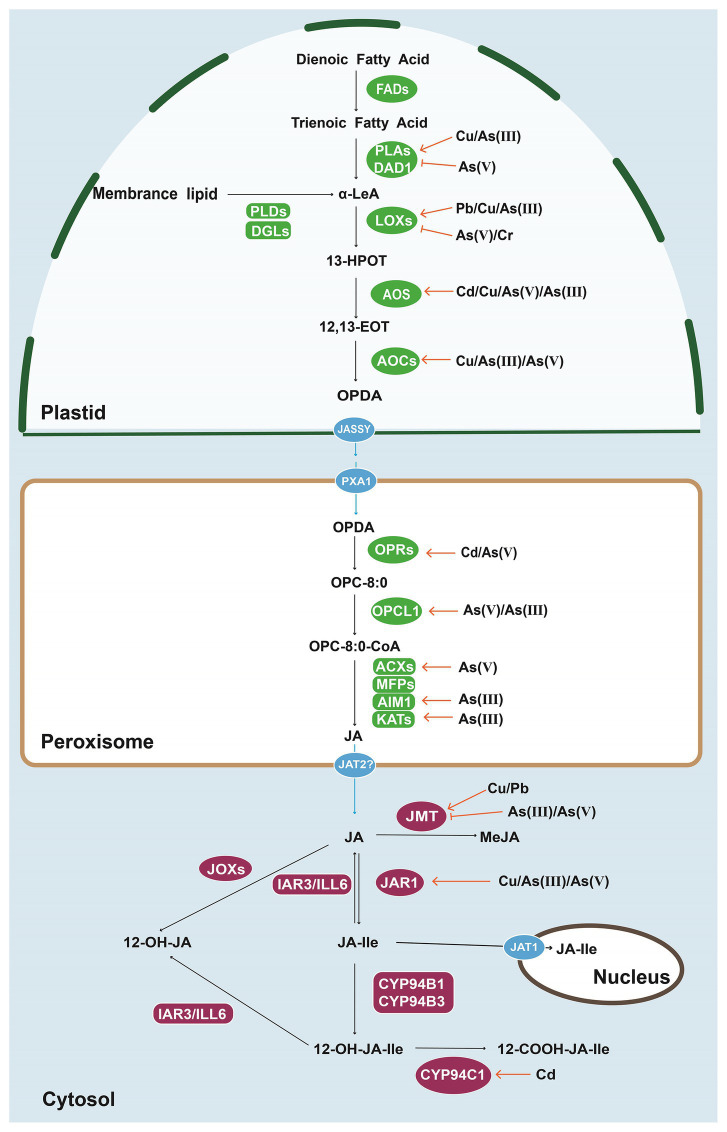
Biosynthesis, metabolism and transport of jasmonic acid (JA) in plant cells. The expression levels of genes encoding enzymes involved in JA synthesis and metabolic pathways are upregulated or downregulated by heavy metals and metalloids [copper (Cu), arsenic (As), cadmium (Cd), chromium (Cr), and lead (Pb)] in rice and *Arabidopsis*. FADs, fatty acid desaturases; PLAs, phospholipases A; DAD1, defective in Anther Dehiscence 1; PLDs, phospholipases D; DGLs, DONGLE, a homolog of DAD1; LOXs, lipoxygenases; AOSs, allene oxide synthases; AOCs, allene oxide cyclases; OPRs, OPDA reductases; OPCL1, OPC-8:0 CoA ligase 1; ACXs, acyl-CoA oxidase; MFPs, multifunctional proteins; AIM1, abnormal inflorescence meristem1; KATs, ketoacyl-CoA-thiolases; JMT, jasmonic acid carboxyl methyltransferase; JOXs, jasmonate-induced oxygenases; IAR3, IAA-Ala-resistant 3; ILL6, IAA-Leu resistant-like 6; JAR1, JA-amino acid synthetase; *α*-LeA, α-linolenic acid; 13-HPOT, 13-hydroperoxylinoleic acid; 12, 13-EOT, 12, 13-(S)-epoxy-octadecanoic acid; OPDA, 12-oxo-phytodienoic acid; OPC-8:0, 3-oxo-2(29-[Z]pentenyl) cyclopentane-1-octanoic acid; OPC-8:0-CoA, 3-oxo-2-(cis-29-pentenyl)-cyclopentane-1-octanoyl CoA; JASSY, A chloroplast outer membrane protein; PXA1, peroxisomal ABC-transporter 1; and JAT, jasmonic acid transporter.

At the molecular level, toxic element-induced JA is largely attributed to the upregulated genes encoding the enzymes for JA biosynthesis. We summarized some key signaling components in Figure 1. For example, the expression levels of *AtLOX3*, *AtLOX4*, and *AtAOS* are rapidly increased in the roots subjected to Cd for 1 h (Lei et al., 2020b). The Cu-increased JA accumulation in rice is likely through the enhanced expression of genes encoding JA biosynthesis-related enzymes such as phospholipase, LOXs, 12-Oxo-PDAreductase (OPR), AOS (Lin et al., 2013). Moreover, transcriptomic analyses reveal that the pathways of JA biosynthesis and signaling are activated in rice roots under As(V) stress (Huang et al., 2012). The expression levels of seven genes including *OsDAD1;2*, *OsDAD1;3*, *OsLOX2;1, OsLOX2;3*, *OsAOS1, OsAOS2*, and *OsAIM1* with putative functions in JA biosynthesis were elevated with As(V) exposure (Huang et al., 2012). Increased levels of *OsJAR1;2* and *OsJAR1;3* for MeJA deactivation where found but significantly decreased transcripts of *OsJMT1*, *OsJMT2*, and *OsJMT4* (Huang et al., 2012) for JA-Ile production suggest that the JA signaling in rice root under As(V) stress is mainly dependent on JA-Ile (Figure 1). Increased expression levels of putative *OsDADs*, *OsLOXs*, *OsAOSs*, and *OsAOCs* are ubiquitously detected in rice plants with the treatments of Cu (Lin et al., 2013), and Cd (Tan et al., 2017), however, more *OsOPRs* are inducible by Cd (Tan et al., 2017). Recently, expression levels of JA biosynthesis genes including *OsLOX1/9/11*, *OsAOS4*, *OsAOC*, *OsOPR1*, and *OsJAR1* have been markedly upregulated in the roots of rice *oswrky28* knockout mutant, indicating the negative role of OsWRKY28 in JA generation. Decreased As concentration is detected in the SH of mutant but it does not relate to changes in the expression of As(V) transporter genes (Wang et al., 2018). Consistently, the JA-deficient mutant plants are more sensitive to heavy metal stress than that of wild type. For instance, the *AtAOS* knockout *Arabidopsis* exhibited more serious chlorosis symptoms and shorter root length with Cd exposure (Lei et al., 2020b). Tomato mutant *suppressor of prosystemin-mediated responses 2* (*spr2*) without chloroplast fatty acid desaturase (FAD) activity display dramatically reduced biomass and increased Cd accumulation due to the severe reduction in JA (Zhao et al., 2016). When exposed to As(III), rice jasmonate-biosynthetic mutant *coleoptile photomorphogenesis 2* (*cpm2*) displays reduced number of adventitious roots and inhibited As(III) root-to-shoot translocation (Ronzan et al., 2019). Therefore, a better understanding of the physiological responses and molecular interactions between JA biosynthesis and toxic minerals may guide future application of JAs in alleviating the toxicity in many food crops and plant species.

## JA Signaling Pathways in Response to Toxic Elements

Although the physiological roles of jasmonates in reducing the toxicity of mineral elements have been demonstrated, the molecular mechanisms on the detoxification and reduced transport and accumulation of toxic elements are unclear. Here, we proposed a putative JA regulatory network in response to heavy metals and metalloids by analyzing the published datasets.

**Figure 2:**
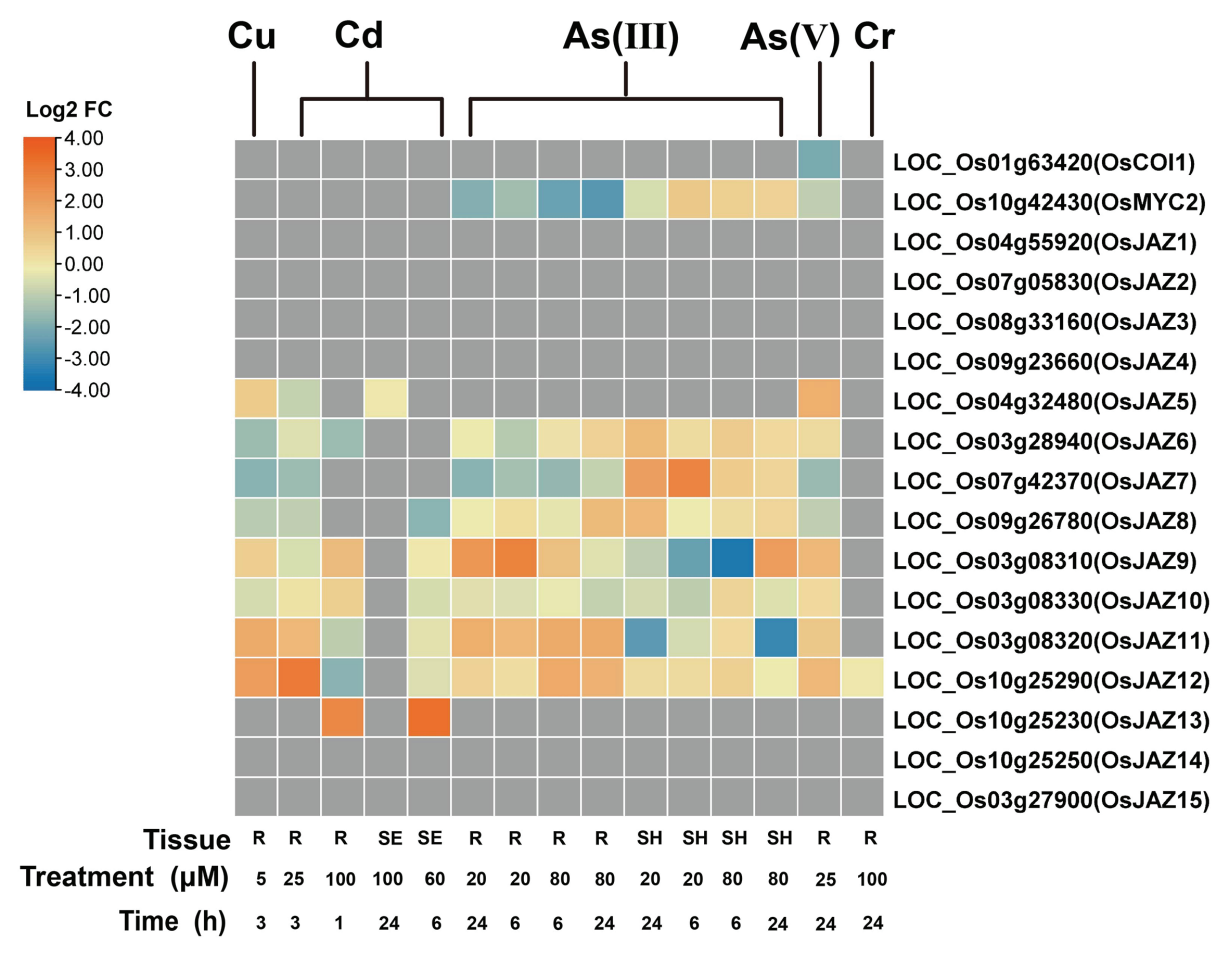
Expression pattern of *OsCOI1*, *OsMYC2*, and *OsJAZs* in response to Cu, Cd, As, and Cr treatments. The heat map was generated with TBtools (Chen et al., 2020), which shows the expression of core JA signaling genes compared with those under the control conditions. Samples are from roots (R), seedlings (SE), and shoots (SH), respectively. The data are displayed as Log2 fold change (Log2 FC). Original data are from Huang et al. (2012), Yu et al. (2012), Lin et al. (2013), Dubey et al. (2014), Tang et al. (2014), and Tan et al. (2017).

Responses of genes encoding proteins consisting of the SCF^COI1^-JAZ complex critical for JA signaling are investigated by using the published transcriptomic datasets of rice (Figure 2). A total of 15 JAZs have been identified in the rice genome (Ye et al., 2009). The transcripts of six genes encoding *OsJAZs* (*OsJAZ5*/6/9/10/11/12) were upregulated by As(V) in rice roots (Huang et al., 2012). Similarly, the expression of *OsJAZs* was elevated in the roots of rice seedlings (SE) subjected to As(III) (*OsJAZ6/8/9/11/12*; Yu et al., 2012), Cd (*OsJAZ6/9/10/11/12/13*; Tang et al., 2014; Tan et al., 2017), and Cu (*OsJAZ5~12*; Lin et al., 2013; Figure 2), but the transcription of *OsJAZ6~12* is inhibited in the rice shoots exposed to As(III) (Yu et al., 2012). A dramatically reduced expression of *OsJAZ9* was observed in the shoots treated with a high concentration of As(III) for 6 h. No significant difference in the expression levels of *OsJAZ1~4* was detected between As(III) stress and control conditions in both roots and shoots (Figure 2). The expression levels of *OsMYC2*, a putative transcriptional factor that directly regulates JA responsible genes, is slightly reduced in the roots but increased in the shoots with As(III) stress (Yu et al., 2012). Further, the abundance of *OsCOI1* transcripts is hardly altered under toxic heavy metals and metalloids (Figure 2; Huang et al., 2012; Yu et al., 2012; Lin et al., 2013; Dubey et al., 2014; Tang et al., 2014; Tan et al., 2017). Although molecular and physiological evidence of these proteins in heavy metals and metalloids have not been elucidated in rice, the results in *Arabidopsis* showed that JA insensitive *AtCOI1* knockout line exhibits severe growth retardation under Cd treatment and cannot be recovered with the application of exogenous MeJA (Lei et al., 2020b), indicating the involvement of AtCOI1 in JA-mediated tolerance to Cd stress.

Plant mineral transporters are critical for the accumulation and detoxification of heavy metal metals and metalloids through uptake, xylem/phloem loading and unloading, as well as sequestration (Sharma et al., 2020; Zhao and Wang, 2020; Deng et al., 2021). Various toxic metal transporters in distinct families have been identified and characterized during the last few decades (Che et al., 2018; Huang et al., 2020; Tang and Zhao, 2021). Recently, it has been revealed that jasmonates coordinate the transport systems of the toxic minerals to restrict accumulation and enhance tolerance (Lei et al., 2020b; Mousavi et al., 2020; Verma et al., 2020). For instance, the expression of the *AtIRT1*, *AtHMA2*, and *AtHMA4* genes responsible for Cd uptake and long-distance translocation from root to shoot is decreased by exogenous MeJA along with reduced Cd accumulation in SE and enhanced tolerance (Lei et al., 2020b). Accordingly, upregulated expression of *HMAs*, as well as the increased Cd content and sensitivity to Cd were detected in JA-deficient mutant *ataos*, which can be restored by the application of exogenous MeJA (Lei et al., 2020b). Pivotal transporters such as OsLsi1 (rice low silicon 1), OsLsi2 (Ma et al., 2008), Nodulin 26-like intrinsic proteins (NIPs; Deng et al., 2020), OsNramp1 (Tiwari et al., 2014), and OsABCC1 (Song et al., 2014; Deng et al., 2018) function in the uptake, root-to-shoot translocation, compartmentation and deposition of arsenite [As(III)] or As(III)-phytochelatins (PCs) complex in rice. MeJA reduced As accumulation in rice by modulating the expression of genes for As(III) uptake (*OsLsi1*, *OsLsi2*, *OsNIP1;1*, and *OsNIP3;1*), translocation and distribution [*OsLsi6*, and Inositol Transporter 5 (*OsINT5*)], as well as detoxification (*OsNRAMP1* and *OsABCC2*; Mousavi et al., 2020; Verma et al., 2020). Although some transporters and genes responsible for the accumulation and detoxification of Cu, Ni, and Pb have been identified (Deng et al., 2013; Du et al., 2015; Fan et al., 2016; Huang et al., 2016; Lange et al., 2017; Garcia de la Torre et al., 2020), the involvement of those transporters in JA-mediated detoxification (Azeem, 2018; Bali et al., 2019) have not been elucidated. Furthermore, the direct transcriptional factors controlling JA-responsive transporter genes are not identified.

The ameliorating effects of jasmonates partially rely on the induced capacity of chelating and reactive oxygen species (ROS) scavenging. The thiol-contained peptides such as glutathione (GSH), PCs, and metallothioneins (MTs) play crucial roles in protecting plants from heavy metals and metalloids stress (Leszczyszyn et al., 2013; Hu et al., 2020; Deng et al., 2021). Both JA and heavy metals induced the transcription of genes for GSH synthesis including *γ-glutamylcysteine synthetase* (*γ-ECS*), *glutathione synthetase* (*GSH*), and glutathione reductase (*GR*; Xiang and Oliver, 1998). Exogenous MeJA increased GR activities and GSH-pools in Cd-stressed rice, leading to reduced Cd uptake and then enhanced Cd tolerance (Singh and Shah, 2014). Similar effects of jasmonates are observed in soybean under Cd stress (Noriega et al., 2012). Cd-induced expression of type-2 metallothionein gene (*KoMT2*) in the leaves of *Kandelia obovata* is restored by exogenous application of MeJA, which leads to the inhibited Cd uptake and root-to-shoot translocation (Chen et al., 2014). On the other hand, production of ROS including hydrogen peroxide (H_2_O_2_) and malondialdehyde (MDA) content in plants is increased significantly by mineral stress, while the activities of classic antioxidant enzymes such as catalase (CAT), peroxidase (POD), superoxide dismutase (SOD), ascorbate peroxidase (APX), and GR can be enhanced by jasmonates for detoxification and promotion of plant growth (Rodríguez-Serrano et al., 2006; Huang et al., 2012; Noriega et al., 2012; Singh and Shah, 2014; Sirhindi et al., 2016; Azeem, 2018; Bali et al., 2019; Mousavi et al., 2020). Furthermore, pretreatment with JA effectively ameliorated Cd-induced oxidative stress through increasing the heme oxygenase activity, but the enhancement can be abolished by irreversible HO-1 inhibitor Zn-protoporphyrin IX. The results indicated that heme oxygenase is also involved in the JA-elevated ROS scavenging capacity responding to heavy metals and metalloids (Noriega et al., 2012). Many transcriptional factors such as AtZAT6 and AtWRKY12 have been identified as activators or repressors of AtGSH1 (Hu et al., 2020), but their regulation by JA still needs to be elucidated. In addition, comparative biochemical and transcriptional profiling has identified differently expressed genes and proteins responsive to heavy metals and metalloids stress (Huang et al., 2012; Yu et al., 2012; Lin et al., 2013; Tang et al., 2014; Kumar et al., 2015; Srivastava et al., 2015; Tan et al., 2017); however, the involvement of these genes in JA-responsive signaling pathways needs to be investigated in the future.

## Regulatory Components of JA-Responsive Signaling Pathways in Response to Toxic Elements

Plant response to heavy metals and metalloids should be integrated into breeding programs to optimize their growth, development, and metabolism for survival. Although large number of functional proteins involved in the accumulation and detoxification of toxic elements have been identified (Clemens and Ma, 2016; Lindsay and Maathuis, 2017; Lei et al., 2020a; Sharma et al., 2020; Zhao and Wang, 2020; Tang and Zhao, 2021), the signal transmission from mineral stress sensing to the regulation of downstream genes is less known. Regulatory models at molecular levels in various plant species have been proposed based on the systematic transcriptomic and biochemical analyses (DalCorso et al., 2010; Huang et al., 2012; Yu et al., 2012; Deng et al., 2020; Wang et al., 2020). Usually, the regulatory networks consist of rapidly activated ROS production and calcium (Ca) oscillation, which can be perceived by Ca-binding proteins and magnified *via* kinases and further downstream pathways such as phytohormones, transport systems, and ROS scavenging, are precisely modulated to induce an appropriately reactive physiological response (Deng et al., 2020).

**Figure 3:**
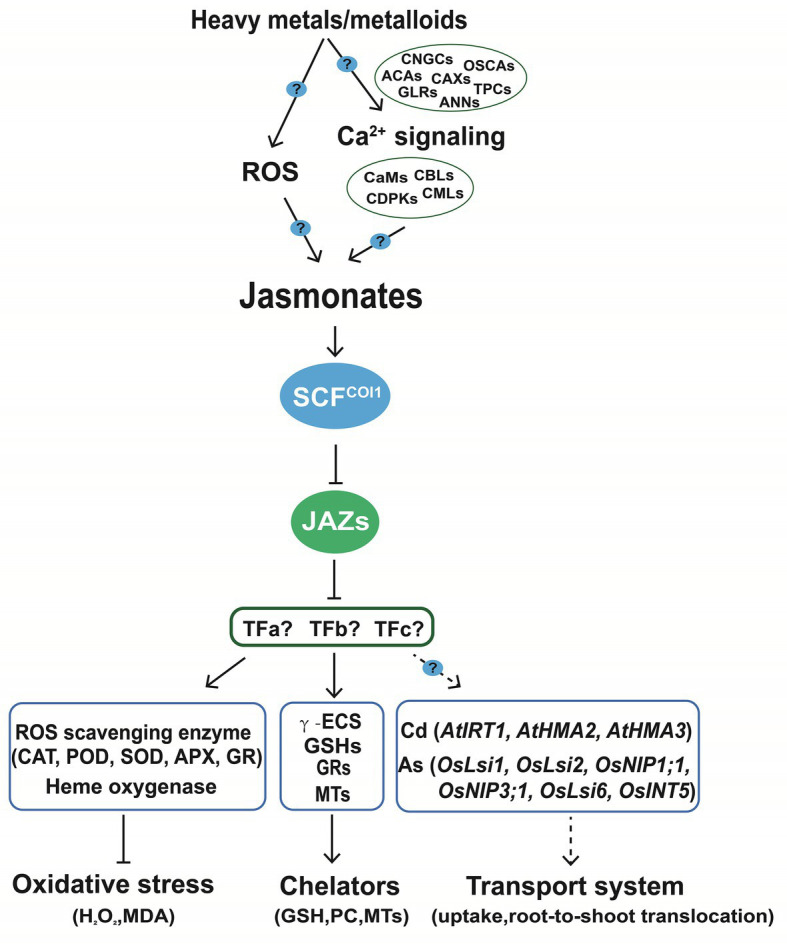
Jasmonic acids limit the accumulation and enhance the tolerance to the toxic elements by coordinating the transport system, activity of antioxidant enzymes, and chelating capacity in plants. Heavy metals and metalloids trigger the generation of JA partially *via* the ROS or Ca^2+^ signaling routes, in which Ca^2+^ channels such as annexins and GLRs may be involved. Active forms of JAs are perceived and transmitted to the downstream targets including secondary transcriptional factors through SCF^COI^-JAZ complex. The enhanced tolerance and reduced accumulation of toxic elements is attributed to the enhanced ROS scavenging activity, chelating capacity, and coordinated transport system. GLRs, glutamate receptor-like proteins; CaMs, calmodulins; CMLs, CaM-like proteins; CBLs, calcineurin B-like proteins; CDPKs, calcium-dependent protein kinases. GSH, glutathione; PCs, phytochelatins; MTs, metallothioneins; *γ*-ECS, γ-glutamylcysteine synthetase; GR, glutathione reductase; CAT, catalase; POD, peroxidase; SOD, superoxide dismutase; and APX, ascorbate peroxidase.

Heavy metals and metalloids are proposed as potent abiotic elicitors for triggering JA accumulation and signaling (Xiang and Oliver, 1998; Maksymiec et al., 2005). We suggest that common components can be found in the JA pathways induced by insect herbivory, toxic minerals, and other abiotic stresses. Here, we highlight that the roles of Ca^2+^ in the mitigation of heavy metals toxicity that may partially rely on activating JA (Figure 3). Ca^2+^ influxes and phosphorylation status are immediately changed when plants are subject to insect attack (Yan et al., 2018a), As(V) (Yu et al., 2012), and Cd (Zhang et al., 2020a). Ca channels and transporters including autoinhibited Ca^2+^-ATPases (ACAs), GLRs, cyclic nucleotide-gated channels (CNGCs), two-pore Ca^2+^ channels (TPCs), the hyperosmolality-gated calcium-permeable channels (OSCAs), Ca^2+^/H^+^ exchangers (CAXs), and annexin proteins (ANNs) are involved in the biosynthesis and signaling of JA. Then the information encoded in the Ca^2+^ signatures can be translated into phosphorylation of specific target proteins for further responses *via* different Ca sensors, including calmodulins (CaMs), CaM-like proteins (CMLs), calcineurin B-like proteins (CBLs), CBL-interacting protein kinases (CIPKs), and calcium-dependent protein kinases (CDPKs; Gao et al., 2018; Wang et al., 2019c; Deng et al., 2020). JAZ-interacting proteins such as MYC2 regulates JA responsive genes (Kazan and Manners, 2013; Howe et al., 2018), but the transcriptional regulators linking JA perception and downstream responses including chelation, ROS scavenging capacity, and mineral transport are not well studied (Figure 3).

## Evolution of Jasmonates-Mediated Detoxification of Heavy Metals and Metalloids in Green Plants

The molecular mechanisms of jasmonate biosynthesis and signaling have been well elucidated in model plants, such as *Arabidopsis* (Howe et al., 2018; Wasternack and Strnad, 2019). Many proteins critical for the detoxification and accumulation of heavy metals and metalloids are also traced to the ancestral green algae (Hu et al., 2020; Deng et al., 2021). This implies the possibility that the regulatory network linking jasmonates and plant responses to toxic metals and metalloids seems to be evolutionarily conserved. Therefore, comparative genetic analyses were conducted to identify and trace the evolutionary history of the key genes and families involved.

### Comparative Genetic and Evolutionary Analysis of Genes in Jasmonates Pathways

Many core components of jasmonate signaling have been identified and the intact signaling pathway is established. Many enzymes participating in the synthesis of JA and conversions from JA to JA-Ile or MeJA are upregulated by the treatments of toxic heavy metals and metalloids (Figures 1–3). The potential orthologs genes with over 20% similarity of the proteins critical for the biosynthesis, metabolism, transport, and signaling are identified through comparative genetic analysis of the genomic datasets from 38 species in three algal and eight land plant lineages (Chen et al., 2017; Adem et al., 2020).

**Figure 4:**
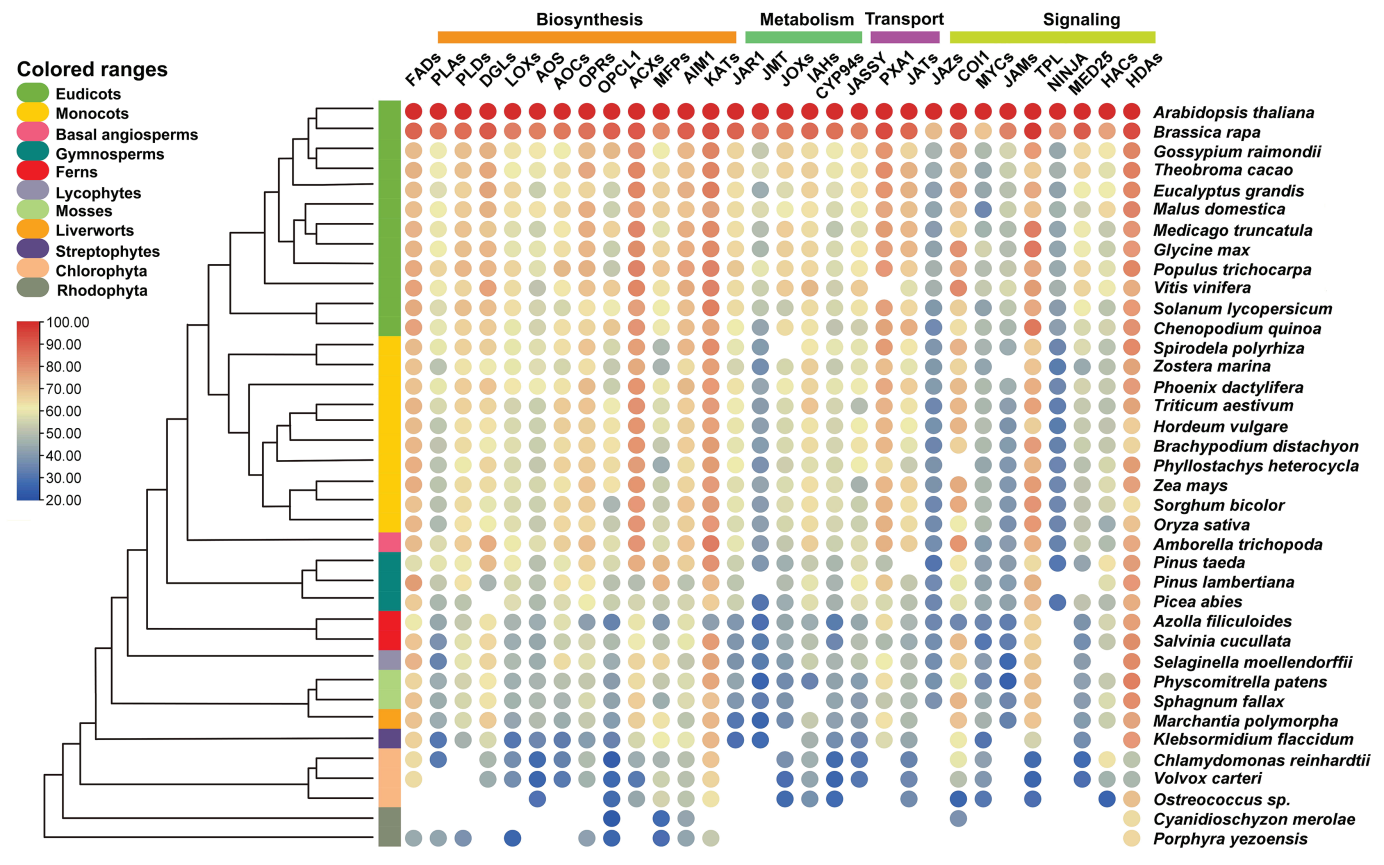
Similarity heat map of JA-related proteins involved in biosynthesis, metabolism, transport, and signaling in different plant and algal species. Candidate protein sequences were selected by BLASTP searches, which satisfied E value <10^−10^ and query coverage >50%. Colored circles indicate protein sequence similarity from 20 (Blue) to 100% (red). The heat map is generated by using TBtools (Chen et al., 2020). Space without colored circles indicates no proteins that satisfied the selection criteria. FADs, fatty acid desaturases; PLAs, phospholipases A; PLDs, phospholipases D; DGLs, DONGLE, a homolog of DAD1; LOXs, lipoxygenases; AOS, allene oxide synthase; AOCs, allene oxide cyclases; OPRs, OPDA reductases; OPCL1, OPC-8:0 CoA ligase 1; ACXs, acyl-CoA oxidases; MFPs, multifunctional proteins; AIM1, abnormal inflorescence meristem 1; KATs, ketoacyl-CoA-thiolases; JMT, jasmonic acid carboxyl methyltransferase; JOXs, jasmonate-induced oxygenases; IAHs, ILR1-like amidohydrolases; CYP94s, cytochrome P450 94s enzymes; JASSY, A chloroplast outer membrane protein with a START domain; PXA1, peroxisomal ABC-transporter 1; JATs, ATP-binding cassette G transporters; JAZs, Jasmonate-ZIM domain proteins; COI1, coronatine insensitive 1; MYCs, bHLH ZIP transcription factors; JAMs, jasmonate-associated MYC2-Like transcription factors; TPL, TOPLESS protein; NINJA, novel interactor of JAZ; MED25, mediator25; HACs, histone acetyltransferases; and HADs, histone deacetylases.

The genetic similarity analysis of the candidate proteins reveals that HDAs display the highest similarity across the green plants, followed by KATs, ACXs, and FADs. The lower similarity of orthologs was found among JAMs, JMT, JAZ, and NINJA (Figure 4). Most orthologs of the enzymes required for the *de novo* synthesis of JA have been identified in the examined land plants and a basal Streptophyte alga *Klebsormidium flaccidum* (Figure 4). For instance, the PpAOS1 and PpAOS2 from the moss *Physcomitrella patens* (Stumpe et al., 2006), MpAOS1 and MpAOS2 from the liverwort *Marchantia polymorpha*, and KfAOS from *K. flaccidum* (Koeduka et al., 2015) exhibit enzymatic properties similar to those of angiosperms despite the different specificities of their substrates (Scholz et al., 2012). Functional analysis of MpAOC in *M. polymorpha* (Yamamoto et al., 2015), as well as PpAOC1 and PpAOC2 from *P. patens* (Stumpe et al., 2010), showed the similar activity and subcellular localization to the AOCs in flowering plants. Consistently, JA has been detected in *K. flaccidum* (Hori et al., 2014), indicating the origin of JA synthesis can be traced to the Streptophyte algae – the sister group of land plants. Only OPDA but not JA is detectable in the moss *P. patens* (Stumpe et al., 2010) despite all the putative enzymes are identified in this moss species, implicating that the putative enzymes consisting of ORPs, OPCL1, ACXs, MFPs, abnormal inflorescence meristem1 (AIM1), and KATs may display diverse function as compared to those in higher plants (Han, 2017).

Four JA transporters have been isolated and functionally characterized in *Arabidopsis* (Figure 1). AtJAT1 (Li et al., 2017b) and AtPXA1 (Theodoulou et al., 2005) belong to the G- and D-subgroup of ABC transporter family, respectively, while AtGTR1 is classified in the subgroup of NPF2s (Saito et al., 2015; Ishimaru et al., 2017), however, JASSY seems independent from the known transporter families (Guan et al., 2019). The absence of JASSY and NPFs (Hu et al., 2020) in red algae (Figure 4) demonstrates that the origin of JA-Ile and OPDA transporters may have arisen from Streptophyte or even earlier from Chlorophyte algae, which is consistent with evolutionary origin the biosynthesis of JAs. The orthologs of AtJAT1 are widely distributed in almost all of the examined species except the two Rhodophytes (Figure 4), confirming our previous analysis using the whole ABC family of 130 members (Hu et al., 2020). JA can be converted to derivatives through the metabolic reactions mediated by different groups of enzymes. The generation of two major active forms, JA-Ile and MeJA, is catalyzed by the enzymes JAR1 and JMTs, respectively (Seo et al., 2001; Staswick and Tiryaki, 2004). It appears that gene families in JA metabolism are less conserved in these examined species compared to those in JA biosynthesis and transport (Figure 4). JMTs for converting JA to MeJA are common in examined angiosperms, gymnosperms, moss *P. patens*, liverwort *M. polymorpha*, and streptophytes *K. flaccidum* (Figure 4). The homologs proteins of JAR1 required for the generation of active JA-Ile are identified in vascular plants and the ancestral streptophyte algae *K. flaccidum* but not the other genomes consisting of rhodophyte, chlorophyte, and streptophyte algae, liverworts, and mosses. These results may indicate that the active forms of JA in these might not be contributed to JA-Ile and/or MeJA. Consistently, the ligands that bind the COI1 receptor in *M. polymorpha* are OPDA isomers but not JA-Ile (Monte et al., 2018).

The core components of JA signaling consist of a co-receptor SCF^COI^-JAZ complex, which employs JA-Ile as the ligand in higher plants (Howe et al., 2018). In *Arabidopsis*, JAZs belong to the TIFY superfamily (Pauwels and Goossens, 2011), while COI1 is an E3 ubiquitin ligase and is a part of an SCF complex (SCF^COI1^; Xie et al., 1998). Interestingly, the comparative genetic analysis showed COI1 is one of the highest conserved proteins among most of the examined species, but JAZs are less conserved, whereby they are missing in most of the algae species except *K. flaccidum* (Figure 4). However, MpCOI1 from *M. polymorpha* is the receptor of OPDA but not JA-Ile resulting from a single residue substitution (Monte et al., 2018), implying the co-evolution of JA biosynthetic mechanism and receptor specificity in vascular plants. There are 13 members of JAZs in *Arabidopsis* (Howe et al., 2018) but only one member in *M. polymorpha*, MpJAZ, which is closer to V-subgroup of AtJAZs including AtJAZ3/4/9 (Monte et al., 2019). MpJAZ displays the wound-induced expression, nuclear localization, interactions with MYCs, as well as hormone-triggered degradation, which is similar to that of JAZs in *Arabidopsis* (Monte et al., 2019). The *MpJAZ* mutant shows severe developmental defects but can be complemented by AtJAZ3, indicating the conserved physiological functions of JAZ in land plants (Howe and Yoshida, 2019; Monte et al., 2019). The diversification and late evolution of JAZs in higher plants may have equipped the genes with additional functions (e.g., abiotic stress tolerance) apart from the common biotic stress responses to wound and insect damage. However, the function and origin of JAZs still require detailed investigations in the future.

**Figure 5:**
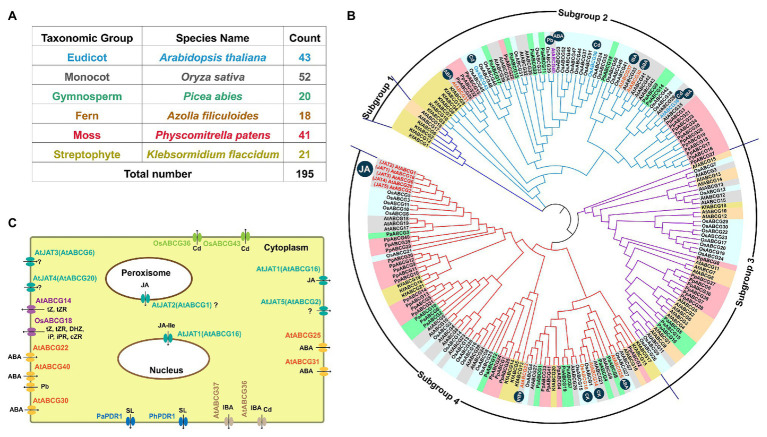
Phylogenetic analysis and phytohormone transport of ATP-Binding Cassette G (ABCG) subfamily. Number **(A)** and phylogenetic tree **(B)** of ABCGs identified in six representative plant species including five land plants and a basal Streptophyte alga. Four major subgroups of ABCGs are shown by different lines. The homologs from the same organism are shaded with the same background color, except the *Arabidopsis* JA transporters (AtJATs). The heavy metal-responsive members including AtABCG36/40, and OsABCG36/43/44 are labeled with black symbols. **(C)** The known subcellular localization of functional characterized ABCGs in various plant species. ABCG homologs with are obtained from the references (Saha et al., 2015; Hwang et al., 2016; Cho et al., 2020) and databases (https://www.uniprot.org/, https://www.fernbase.org/, https://congenie.org/, and http://www.plantmorphogenesis.bio.titech.ac.jp), the phylogenetic tree is generated by using MEGA7 (Kumar et al., 2016). At, *Arabidopsis thaliana*; Os, *Oryza sativa*; Mt, *Medicago truncatula*; Kf, *Klebsormidium flaccidum*; Af, *Azolla filiculoides*; Pa, *Picea abies*; Pp, *Physcomitrella patens*; PhPDR1, *Petunia hybrida* pleiotropic drug resistance 1; tZ, trans-zeatin; tZR, trans-zeatin riboside; cZ, cis-zeatin; cZR, cis-zeatin riboside; iP, isopentenyladenine; iPR, isopentenyladenosine. SL, strigolactone; IBA, indole-3-butyric acid; ABA, abscisic acid; JA, jasmonic acid; and JA-Ile, jasmonoyl-isoleucine.

The involvement of ATP-Binding Cassette G (ABCG) transporter proteins in both JA transport and heavy metal detoxification has led us to explore whether there are any links by further analyses of the ABCG subfamily using six representative plant species. We obtained 21, 41, 18, 20, 52, and 43 potential members in *K. flaccidum*, *P. patens*, the fern *Azolla filiculoides*, *Picea abies*, rice, and *Arabidopsis*, respectively (Figure 5A). The 195 ABCG proteins can be classified into four subgroups (Figure 5B). ABCGs are multifunctional transporters employing both phytohormones (Kretzschmar et al., 2012; Sasse et al., 2015) and heavy metals as substrates. The heavy metals-responsive ABCGs including AtABCG36 (Kim et al., 2007), AtABCG40 (Lee et al., 2005), OsABCG36 (Fu et al., 2019), OsABCG43 (Oda et al., 2011), and OsABCG44 and are mainly in Subgroup 2 (Figure 5B). The plasma membrane-localized Cd efflux transporters AtABCG36 (Strader and Bartel, 2009) and AtABCG37 (Ruzicka et al., 2010) function as indole-3-butyric acid (IBA) transporters too (Figure 5C). Likewise, the Pb efflux pump AtABCG40 is also an abscisic acid (ABA) uptake transporter (Kang et al., 2010). Three additional transporters AtABCG25, AtABCG31, and AtABCG30 cooperatively facilitate ABA from the endosperm to the embryo to repress seed germination together with AtABCG40 (Figure 5C; Kang et al., 2010, 2015). The other ABA transporter AtABCG22 is required for stomatal regulation (Figure 5C; Kuromori et al., 2011). AtABCG14 (Ko et al., 2014; Zhang et al., 2014) and OsABCG18 (Zhao et al., 2019b) are essential for the root-to-shoot translocation of cytokinins including trans-zeatin, trans-zeatin riboside. The hormone transporters, AtABCG22, AtABCG25, and jasmonic acid transporters (JATs) are mainly located in subgroup 4 (Figure 5B). Given the close phylogenetic relationships of ABCG orthologs that have functions in the transport of heavy metals (Pb and Cd) and ABA and IBA in Subgroup 2, it would be interested to explore the ABCGs in Subgroup 4 that contain unique JATs and putative transporters for heavy metals and metalloids in the future.

**Figure 6:**
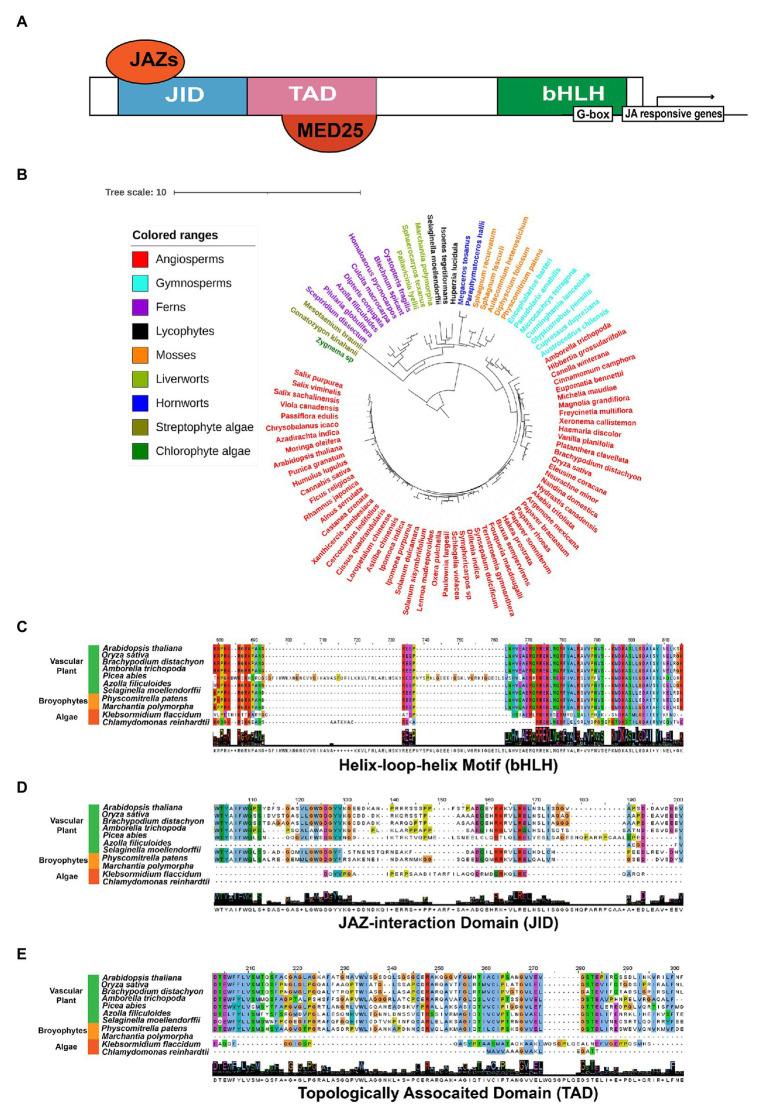
Evolutionary conservations and diversity of MYC2s orthologs in green plant species **(A)** simplified schematic diagram of the AtMYC2 protein and the conserved domains. JID, TAD, and bHLH are functional domains interacting with JAZs, Mediator 25 (MED25), and the cis-element G-box in the promoters of JA-responsive genes, respectively. **(B)** Phylogenetic trees of MYC proteins in representative species of major lineages of green plants using OneKP database. The tree is generated using the maximum-likelihood method. Clades are indicated by different colors. Alignment of the bHLH **(C)**, JID **(D)**, and TAD **(E)** domains among 11 representative green plant species. JID, JAZ-interaction domain; TAD, topologically associated domain; bHLH, basic helix–loop–helix motif; and JAZs, jasmonate-ZIM domain proteins.

MYCs belong to the IIIe-subgroup of bHLHs, which have been demonstrated as the primary transcriptional factors inducing the expression of JA response genes (Kazan and Manners, 2013; Zander et al., 2020). The typical MYC proteins consist of three functional domains, JAZ-interaction Domain (JID), Topologically Associated Domain (TAD), and bHLH (Figure 6A). JID and TAD are located in the N-terminal region of the protein and responsible for the interaction of JAZs, and the binding and transactivation of MED25, respectively. Additionally, bHLH is required for heterodimerization and binding to the G-box sequence in target promoters (Kazan and Manners, 2013; Figure 6A). In our results, the putative MYCs are found in all land plants, the streptophyte alga *K. flaccidum* and three Chlorophyte algae (Figures 4, 6B). Using the key member AtMYC2 (Zander et al., 2020) as our search query, we obtained 953 orthologs from the OneKP database (Figure 6B; One Thousand Plant Transcriptomes Initiative, 2019). Sequence alignment analyses exhibited highly conserved bHLH domain of the MYCs in the representative species of the major green plant lineages, suggesting a potential early evolution of bHLH domain in chlorophyte algae (Figure 6C). Consistent with the evolution of JAZs, JID domains are found to be less conserved in the selected green plants, indicating the JAZ-JID signaling may have diversified for multiple functions in biotic and abiotic stress response in higher plants (Figure 6D). Histone acetyltransferase encoding by *HAC1* is an activator of MYC2-regulated transcription through interaction protein of MED25 (Wang et al., 2019b), a subunit of conserved multi-subunit co-regulatory complex essential for Pol II-dependent transcription in eukaryotic cells (An et al., 2017). Moreover, TAD motifs are absent in *M. polymorpha* and less conserved in the two algal species (Figure 6E), despite the putative interacting MED25 proteins, which can be observed in *M. polymorpha*, algae species *K. flaccidum*, *Chlamydomonas reinhardtii*, and *Volvox Carteri* (Figure 4). The predicted origin of HAC orthologs is analogical to that of MED25, implicating the possibility of co-evolution of the two proteins. However, the protein-protein interactions and evolution between MYCs and the putative candidate genes need to be validated in some key species. JAMs negatively regulate the JA responses as the competitors of MYC2 in *Arabidopsis* (Sasaki-Sekimoto et al., 2013). Different from the widely presented of bHLHs in land plants and Streptophytes (Hu et al., 2020), JAMs belong to the IIId-subgroup of bHLHs and are seed plant-specific (Figure 4). The putative co-repressor TPLs are presented in all examined green plants, while the bridge protein NINJAs is mainly found in land plants (Figure 4), which are consistent with the previous analyses (Han, 2017). Moreover, the potentially epigenetic regulators HDAs show very high similarity among 36 detected species (Figure 4). In summary, the 30 gene families encoding biosynthesis, metabolism, transport, and signaling of JA and its derivatives are found in most tested land plants and are originated from the basal streptophyte algae (26 out the 30 gene families). However, the level of sequence similarity and conservation vary largely among the gene families. These analyses indicate that the function of the orthologs of SCF^COI1^-JAZ-MYC complex in JA signaling may be the fundamental machinery required for adaptation to the terrestrial environment and its associated presence of heavy metals and metalloids.

### Linking JA Signaling to Transport and Detoxification of Heavy Metals and Metalloids

Jasmonic acid-mediated decreased accumulation and detoxification of heavy metals and metalloids is largely dependent on the transport system, antioxidant effect, chelation, and sequestration functions. Our previous analyses reveal that homologs of ZIPs including AtIRT1 critical for Cd uptake were identified in almost all examined green plants (Hu et al., 2020). HMAs for Cd/Zn transporting and PHTs for As(V) allocation, NIPs for As(III) mobility, ABC transporters for GSH- or phytochelatins (PCs)-conjugated heavy metal detoxification and sequestration represent the early evolution from ancestral algae (Deng et al., 2021). The putative NIPs are consistent with the finding that NIPs originate from horizontal gene transfer of bacterial aquaporin group with As efflux activity (Pommerrenig et al., 2020). Genes encoding putative glutathione synthetases (GSH1 or *γ*-ECS, GSH2 homologs) can be traced to an ancestral streptophyte alga *K. flaccidum*, however, the potential phytochelatin synthetase (PCS) orthologs for PCs generation are mainly presented in vascular plants (Hu et al., 2020). ROS play multiple beneficial roles at low concentrations, but cause cellular damage through oxidative stress at high concentrations. ROS are the byproducts of aerobic metabolism, the homologs of enzymes for ROS scavenging and signaling are evolutionarily conserved among all examined land plant species and the ancestor chlorophyte algae (Zhao et al., 2019a).

Calcium signaling is involved in JA regulatory network and also plays important roles in the transmission of the signals generated by heavy metals and metalloids stress to physiological responses (Zhang et al., 2020a). Comparative genomic and evolutionary studies reveal the widespread occurrence of channels, pumps, and transporters likely to be involved in Ca signaling (Verret et al., 2010; Edel et al., 2017; Thor et al., 2020). Putative ACAs and TPCs have been identified in red algae *Porphyra yezoensis*, the number of ACA members is rapidly expanded in land plants since the arise of ACAs of green algae *C. reinhardtii*, however, the members of TPCs are likely to be reduced in the examined of higher plants (Cai et al., 2017; Chen et al., 2017). CNGCs and GLRs are generally present in land plants and green algae, furthermore, isoforms of GLRs but not CNGCs have been found in seaweed *P. yezoensis* and brown alga *Ectocarpus siliculosus* (Cai et al., 2017; Chen et al., 2017). Furthermore, The CNGC family have been greatly expanded in seed plants, while increased number of GLRs is emerging since the arise of aquatic fern species *Salvinia cucullata* and *A. filiculoides* (Verret et al., 2010; De Bortoli et al., 2016; Cai et al., 2017). However, canonical CNGC does not exist in unicellular algae species including *Ostreococcus lucimarinus*, *V. carteri*, and *C. reinhardtii* because the lacking of plant CNGC-specific motif (De Bortoli et al., 2016). Moreover, further alignment of functional domains reveals the common residues responsible for ion selectivity and gating among land plant glutamate receptors are different to algae (De Bortoli et al., 2016). ANNs are suggested as a novel type of Ca^2+^ channel, the homologs are also widely present in Chlorophyta green algae, Bryophyta, Lycophyta, and vascular plants, besides, two domains containing well-conserved calcium-binding sites have been identified in many plants (Clark et al., 2012). Homologs of CAXs are widely observed in most of the examined plant species; moreover, there has been an expansion and diversification of CAX family within flowering plants (Emery et al., 2012). Furthermore, protein similarity analyses reveal that they are highly conserved in seed plants (Cai et al., 2017; Chen et al., 2017). The Ca-dependent channel (DUF221) domain-containing OSCAs are conserved across eukarryotes. Phylogenetic analysis of OSCAs reveals four clades of land plant homologs, homologs from the moss *P. patens*, and the spikemoss *Selaginella moellendorffii* are classified into the clade comprising of osmotic-responsive AtOSCA1.2 (Hou et al., 2014), indicating the possibly conserved functions among land plants.

The Ca sensors CaMs are well conserved in eukaryotes, whereas CMLs are mainly found in land plants and algae, however, the number of genes of the two families are not directly linked to the genome size of the organism (Mohanta et al., 2017). The genetic similarity of the Ca dependent protein kinases including CBLs, CDPKs, and CIPKs is higher than in land plants and streptophyte algae, and the value in chlorophyte algae is still higher than 30% (Edel et al., 2017; Zhao et al., 2019a), indicating the extremely early origination of Ca signaling. Furthermore, the diversity and abundance of calcium-signaling components are increased at a far greater rate than general genomic expansion (Marchadier et al., 2016; Edel et al., 2017). CBLs and their interacting partners CIPKs families have been expanded multiple times during the evolution of plants, resulting from retrotransposition, tandem duplication, and whole-genome duplication (Kleist et al., 2014). Most recent studies reveal that the highly specific interaction, together with asymmetric expression patterns to overcome the relatively imbalanced duplicates of CIPKs and CBLs (Zhang et al., 2020b).

## Conclusion

In summary, heavy metals and metalloids elevate endogenous JA levels to alleviate the toxicity possibly through Ca-mediated signaling, enhanced ROS scavenging capacity, chelation activity, and coordinated transport systems (Figures 1–4). The origin of both JA regulated downstream responses to toxic metals and the putative upstream regulators are most likely in parallel with the arising of JA biosynthesis and metabolism since Streptophyte algae – the sister clade of land plants (Figures 4–6). We reviewed pieces of information linking JA signaling and the detoxification of heavy metals and metalloids that are suggested to be the priorities in future research work. These are: (1) identification of the critical Ca channels and sensors responsible for the toxic mineral-induced JA production, (2) discovery of the key transcriptional factors directly regulating downstream genes of the toxic mineral-induced JA production, (3) investigation the functional conservation and diversity of the heavy metal and metalloid stress-related and JA-responsive components *via* genetic complementation in evolutionarily important model green plants such as *Arabidopsis*, rice, moss (*P. patens*), and algae (*K. flaccidum*), in addition, investigation of the conservation and diversity of metal accumulation-induced JA in defense signaling in various hyperaccumulators. The proposed research will shed light on the understanding of the molecular mechanisms of JA signaling and element tolerance, as well as the practices for mitigation of contamination or pollution caused by heavy metals and metalloids. The application of exogenous JA and the derivatives in crops trends to diminish the ingestion of toxic metals and metalloids *via* the food chain, while JA antagonists are candidates for phytoremediation by promoting accumulation activity of plants.

## Author Contributions

FD and Z-HC conceptualized the review. XC performed transcriptomes and comparative genomics analyses, and prepared all the figures together with WJ, TT, and GC. FD, XC, and Z-HC analyzed the results and wrote the manuscript with support from FZ, SJ, WG, ZL. XC, FD, MM, and Z-HC did final editing of the manuscript. All authors contributed to the article and approved the submitted version.

### Conflict of Interest

The authors declare that the research was conducted in the absence of any commercial or financial relationships that could be construed as a potential conflict of interest.
